# Dual Block Copolymer Morphologies in Ultrathin Films on Topographic Substrates: The Effect of Film Curvature

**DOI:** 10.3390/polym14122377

**Published:** 2022-06-12

**Authors:** Elisheva Michman, Meirav Oded, Roy Shenhar

**Affiliations:** Institute of Chemistry and the Center for Nanoscience and Nanotechnology, The Hebrew University of Jerusalem, Jerusalem 9190401, Israel; elisheva.michman@mail.huji.ac.il (E.M.); meirav.oded@mail.huji.ac.il (M.O.)

**Keywords:** block copolymers, directed self-assembly, thin films, hierarchical structures, patterning

## Abstract

The ability to create mixed morphologies using easily controlled parameters is crucial for the integration of block copolymers in advanced technologies. We have previously shown that casting an ultrathin block copolymer film on a topographically patterned substrate results in different deposited thicknesses on the plateaus and in the trenches, which leads to the co-existence of two patterns. In this work, we highlight the dependence of the dual patterns on the film profile. We suggest that the steepness of the film profile formed across the plateau edge affects the nucleation of microphase-separated domains near the plateau edges, which influences the morphology that develops on the plateau regions. An analysis of the local film thicknesses in multiple samples exhibiting various combinations of plateau and trench widths for different trench depths enabled the construction of phase diagrams, which unraveled the intricate dependence of the formed patterns not only on the curvature of the film profile but also on the fraction of the film that resides in the trenches. Our analysis facilitates the prediction of the patterns that would develop in the trenches and on the plateaus for a given block copolymer film of known thickness from the dimensions of the topographic features.

## 1. Introduction

Directed self-assembly (DSA) of block copolymers (BCPs) is one of the leading approaches in the fabrication of nanopatterned substrates and bears great potential for advances in nanoelectronics and nanophotonics [1,2,3,4]. Since its inception over twenty years ago, DSA has progressed from affording highly aligned domains of stripes or dots [5,6,7,8,9,10,11] to facilitating a variety of complex and irregular morphologies [12,13,14,15,16,17,18,19,20,21,22,23,24,25,26,27,28]. The most common DSA approaches rely on chemoepitaxial and graphoepitaxial growth of BCP domains on lithographically patterned substrates [29]. The chemoepitaxial approach utilizes two-dimensional, chemically patterned substrates to orient the BCP domains [5,7,30]. The preparation of chemically patterned substrates requires a few fabrication steps, which involve lithography and chemical modification of specific regions of the substrate. Graphoepitaxial alignment employs topographic features etched into the substrate by a combination of lithographic patterning and pattern transfer [6,8,10,31,32]. This approach lends itself to complex and layered structures due to its three-dimensional nature [12,16,33].

To date, most works on graphoepitaxial DSA focused on orienting BCP domains in films that span at least one BCP period in thickness [34,35]. We have recently found that applying the graphoepitaxial DSA approach to ultra-confined films on selective substrates affords a controllable way to obtain dual patterns with non-bulk morphologies [36]. This study, performed on a lamellar polystyrene-*block*-poly(methyl methacrylate) (PS-*b*-PMMA), revealed that the substrate topography can be used to induce different film thicknesses on the plateaus and in the trenches during the spin coating process. Coupled with the extreme sensitivity of the morphology of ultraconfined films to film thickness and substrate selectivity, this mechanism leads to the formation of different patterns on the plateaus and in the trenches. A follow-up study has substantiated this effect on a different type of block copolymer (polystyrene-*block*-poly(2-vinyl pyridine) with a cylindrical composition) [37].

**Figure 1:**
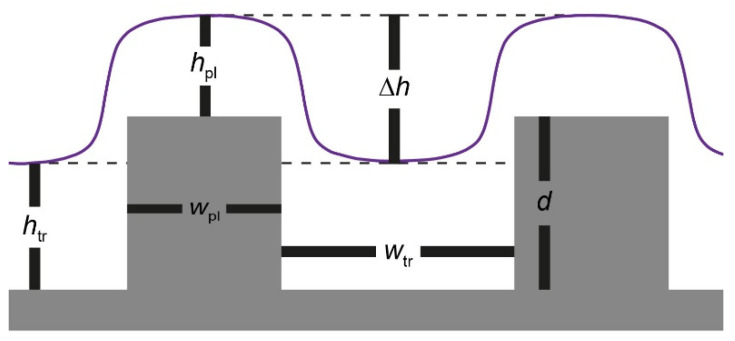
Illustration of the profile of a film coated on a topographic substrate showing the variables used in this study: local films thicknesses on the plateaus and in the trenches (*h*_pl_ and *h*_tr_, respectively), plateau and trench widths (*w*_pl_ and *w*_tr_, respectively), trench depth (*d*), and height contrast (∆*h*).

Here, we expand the research and describe the results of a fundamental study that shows that the dual patterns are influenced not only by the local film thickness and substrate selectivity but also by the trench depth, the lateral feature dimensions, and the interplay between them (Figure 1). Our analysis highlights the role of another variable, which seems to be a dominant factor in the resulting morphology: the profile of the film above the edges of the topographic features.

## 2. Materials and Methods

Materials. PS-*b*-PMMA diblock copolymer (*M*_n_ 312 kDa, PDI 1.27, *f*_PS_ = 0.48, *L*_0_ = 84 nm) was synthesized by standard anionic polymerization under nitrogen atmosphere. The molecular weight, size distribution, and PS weight fraction were determined by gel permeation chromatography (GPC) in tetrahydrofuran against PS standards for the PS block and comparison of the ^1^H NMR signals for the phenyl and methoxy groups for the PMMA block. *L*_0_ was determined by small angle X-ray scattering (SAXS).

Sample preparation. Topographically patterned substrates with varying feature sizes were prepared using electron beam lithography (Raith eLINE, Dortmund, Germany) on silicon wafer substrates using 250 nm-thick poly(methyl methacrylate) (PMMA) resist (495 kDa, Kayaku Advanced Materials, Westborough, MA, USA) followed by cold development (2 min, −5 °C) in MIBK:IPA (1:3) developer solution (Kayaku Advanced Materials, Westborough, MA, USA) and reactive ion etching with C_4_F_8_ and SF_6_ (Oxford Instruments Plasmalab System 100, Bristol, UK). Following etching, substrates were cleaned with acetone, oxygen plasma (Pico plasma asher, Diener, Ebhausen, Germany), and piranha solution. 

Block copolymer thin films were prepared on patterned substrates by spin coating a toluene solution of the BCP (0.9% *w/w*) for 30 s at 3000 rpm, resulting in films with nominal film thickness of 22–23 nm. All films were annealed for 15 min in a closed Petri dish with saturated chloroform vapor environment at ambient temperature.

Characterization. Trench depths were measured using scanning force microscopy (SFM) on a Dimension Icon XR Scanning Probe Microscope (Bruker Corporation, Billerica, MA, USA). Film characterization was performed using high-resolution scanning electron microscopy (FEI Sirion HR-SEM, Hillsboro, OR, USA) and SFM. Local film thicknesses above the trenches and plateaus were determined by scratching away part of the film with a 19 gauge syringe needle, followed by SFM scanning and analysis of the seam between the intact BCP film and the exposed silicon substrate using the step analysis tool (Nanoscope Analysis Program v. 2.0, Bruker Corporation), which averages height values of different scan lines of selected areas. These thicknesses were determined by referencing the SFM height values of the film to the corresponding, completely exposed features, as shown previously [36]. Film height contrasts were measured from the highest part of the film deposited on the plateau to the lowest part of the film deposited in the trench. The first derivatives of the film contour were calculated from neighboring points in the SFM raw data.

## 3. Results

A toluene solution of the block copolymer was spin-coated on topographically defined substrates to yield films with the same nominal film thicknesses of 22–23 nm. All samples featured the same array of alternating stripe patterns, and each sample was etched to form trenches of a fixed depth in the range of 8–38 nm. The plateau widths varied in the range of 160 to 640 nm (~2–8 *L*_0_), and the trench widths varied in the range of 160 to 2000 nm. All films were annealed in a saturated chloroform vapor environment for 15 min at ambient temperature. Chloroform was selected for solvent annealing because it is rather nonselective toward PS and PMMA ($\chi_{{\mathrm{CHCl}}_{3},\mathrm{PS}}$ and $\chi_{{\mathrm{CHCl}}_{3},\mathrm{PMMA}}$ values calculated using Hansen solubility parameters equal to 0.47 and 0.50, respectively) [38].

### 3.1. Dependence on Trench Depth

**Figure 2:**
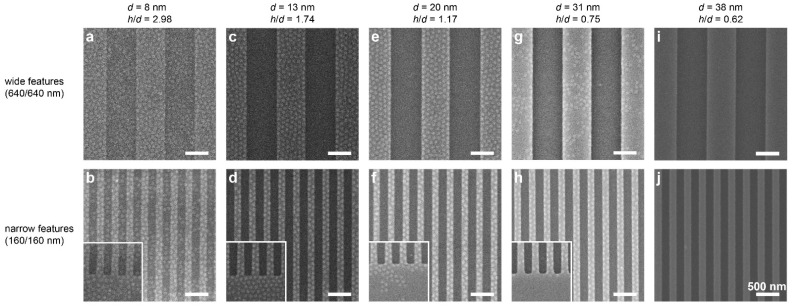
SEM images of substrates with 22–23 nm nominal (cast) film thicknesses on substrates of increasing trench depth: 8 (**a**,**b**), 13 (**c**,**d**), 20 (**e**,**f**), 31 (**g**,**h**), and 38 nm (**i**,**j**). The trench/plateau widths are 640/640 nm and 160/160 nm in the top and bottom row panels, respectively. Bright and dark stripes correspond to the plateaus and trenches, respectively. Insets show the border of the patterned area. All scale bars represent 500 nm. The images shown in panels (**c**,**f**) were adapted with permission from Ref. [36] (copyright 2019 American Chemical Society).

Figure 2 shows scanning electron microscopy (SEM) images of films cast on substrates featuring variable trench depths and symmetric plateau/trench widths, both wide (640 nm) and narrow (160 nm). In the shallowest trench depth, 8 nm, Figure 2a,b, the film displays surface patterns of hexagonally packed dots over both plateaus and trenches, despite the symmetric composition of the BCP. This dot surface pattern has previously been shown to represent neck-shaped PMMA domains resulting from the extreme confinement of the film and the selectivity of the substrate toward PMMA, which depletes the PMMA from the free surface of the film [36]. The dot pattern is visible over the entire substrate surface, both in the trenches and on the plateaus. This behavior is consistent with previous studies of DSA with topographically patterned substrates, which showed that a minimal topographic depth is required to obtain domain alignment [6,35,39].

Films cast at the same nominal thickness (22–23 nm) on substrates featuring deeper trenches (13 and 20 nm) display a different behavior. These films, which are still nominally thicker than the trench depth, display a dot pattern only on the plateaus, whereas the films deposited in the trenches appear patternless, representing a local morphology of lying lamellae (Figure 2c–f). These results are consistent with our results from the previous study, which attributed the lying lamellae in the trenches to the slightly higher local film thickness in them compared to the film thickness on the plateaus [36]. This dual morphology seems rather insensitive to the lateral dimensions of the features, resulting in dual patterns with sharply defined borders. Additionally, it is interesting to note that the emergence of dual patterns occurs already for trench depths that are as low as 0.15 *L*_0_. Kramer et al. [6] and Russell and coworkers [34,35,39,40] have found that the minimal trench depth required for obtaining long-range ordering in films that are thicker than the topographic contrast of the substrate is considerably larger (0.3–0.5 *L*_0_). This suggests that the influence of topography on the formation of dual patterns in ultrathin films is different from its influence on aligning BCP domains in thicker films. In the case of dual pattern formation, it seems the trench depth should be related to the nominal film thickness rather than to the BCP periodicity (i.e., the film thickness defines the relevant length scale). Ross et al., who investigated the alignment of cylinder-forming BCPs using topographic features, also came to the conclusion that the deciding factor governing domain orientation is the ratio of trench depth to film thickness [17].

Interestingly, as the trench depth is increased to 31 nm (i.e., deeper than the nominal film thickness), the dots on the wide plateaus become sparse (Figure 2g), and only narrow plateaus retain a dense array of dots (Figure 2h). For the deepest trenches (38 nm), the pattern is observed neither in the trenches nor on the plateaus (Figure 2i,j). Additionally, images taken close to the border of the pattern (insets in Figure 2b,d,f,h) show a similar trend, where the dots appearing near the border of the topographically patterned area become sparse and are absent in films cast on substrates featuring deeper trenches. It should be noted that although the local film thicknesses in the trenches are lower than the trench depths in these systems, the film profile continues smoothly from the plateau to the trench without dewetting from the trench walls (see Supplementary Figure S1).

The results shown above demonstrate a clear dependence of the morphology on the trench depth. These results are analyzed in terms of the relation between the nominal film thickness, *h*, and the trench depth, *d*. For the shallowest trenches (8 nm; *h* ≈ 3*d*), the film is much thicker than the trench depth, which is apparently below the minimum depth required for inducing dual pattern formation [6,34]. As the trenches become deeper (13 and 20 nm, corresponding to *h* = 1.74*d* and *h* = 1.17*d*), the influence of the topography becomes noticeable, and the morphologies obtained on the plateaus and in the trenches are differentiated by the local film thickness, as we have previously shown [36]. However, as the trenches become even deeper (31 and 38 nm, corresponding to *h* = 0.75*d* and *h* = 0.62*d*, which is deeper than the film thickness), the effect of trench depth on the BCP morphology that develops on the plateaus could not be related to the local film thicknesses alone.

### 3.2. Influence of the Plateau and Trench Widths

As Figure 2g,h shows, a dependence of the surface pattern on the width of the plateau emerges under certain conditions. In this section, we elaborate on this dependence by probing different relationships between plateau and trench widths.

**Figure 3:**
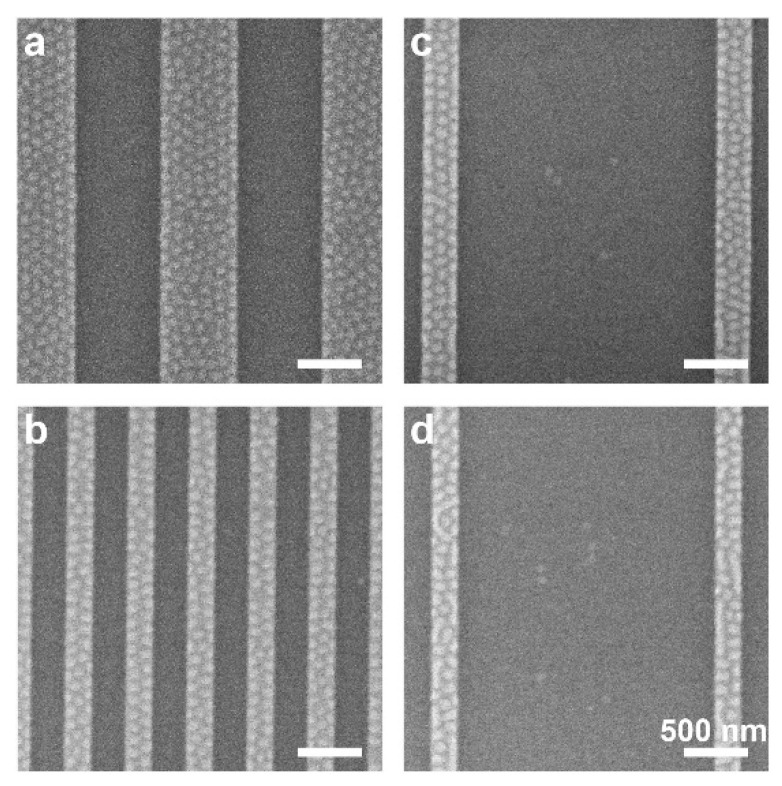
(**a**–**d**) SEM images of films cast on substrates featuring 20 nm-deep trenches, showing patternless trenches and dot-patterned plateaus. Nominal film thickness is 23 nm. Trench/plateau lateral widths: (**a**) 640/640, (**b**) 240/240, (**c**) 2000/320, and (**d**) 2000/240 nm. Bright and dark stripes correspond to the plateaus and trenches, respectively. All scale bars represent 500 nm. The image shown in panel (**c**) was adapted with permission from Ref. [36] (copyright 2019 American Chemical Society).

**Figure 4:**
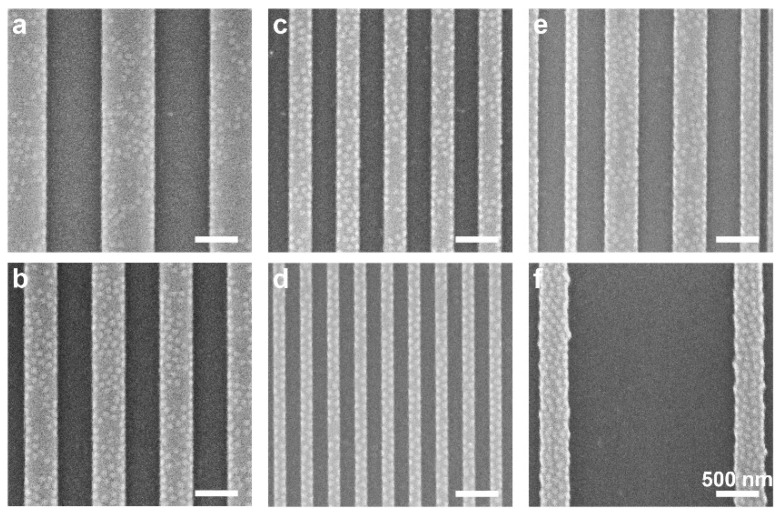
(**a**–**d**) SEM images of films cast on substrates featuring 31 nm-deep trenches and equal plateau and trench widths: (**a**) 640 nm; (**b**) 400 nm; (**c**) 280 nm; (**d**) 160 nm. Nominal film thickness is 23 nm. Bright and dark stripes correspond to the plateaus and trenches, respectively. (**e**) Image of plateaus and trenches with varying lateral dimensions. (**f**) Image of wider (2 µm) trench with narrow 320 nm plateau. All scale bars represent 500 nm.

Figure 3 shows that for the 20 nm-deep trenches, the morphology is largely independent of the lateral dimensions of the plateaus and trenches and the ratio between them, and shows well-defined areas of plateaus covered with dots and patternless trenches. This conclusion was already made above in relation to Figure 2e,f, and is now also exemplified with asymmetric structures, which consist of wide trenches and narrow plateaus. For deeper trenches, however, a dependence of the local morphology on the trench width emerges. Figure 4 shows the morphologies obtained on samples featuring 31 nm-deep trenches that were cast with BCP films with the same nominal film thickness as the other samples (22–23 nm). For the widest plateau sample, measuring 640 nm in width (Figure 4a), the plateau exhibits a partial dot pattern where some areas remain patternless. As the plateau width decreases, the fraction of the plateau area exhibiting the dot pattern increases (Figure 4b–d). The 160 nm-wide plateaus (Figure 4d) are completely covered by the dot pattern, similar to the narrow plateaus with the 20 nm trench depth (Figure 3b). Interestingly, this observed phenomenon depends strongly on the local feature dimensions: Figure 4e shows various plateau widths in close proximity, where the morphology on each plateau is unaffected by the morphology in adjacent plateaus. For very wide trenches (2 µm), the wide trench causes even slightly wider plateaus (320 nm) to be completely covered by the dot pattern (compare Figure 4c, where 280 nm-wide plateaus are partially covered in dots, and Figure 4f, where 320 nm-wide plateaus are completely covered with dots when the adjacent trench is wider).

**Figure 5:**
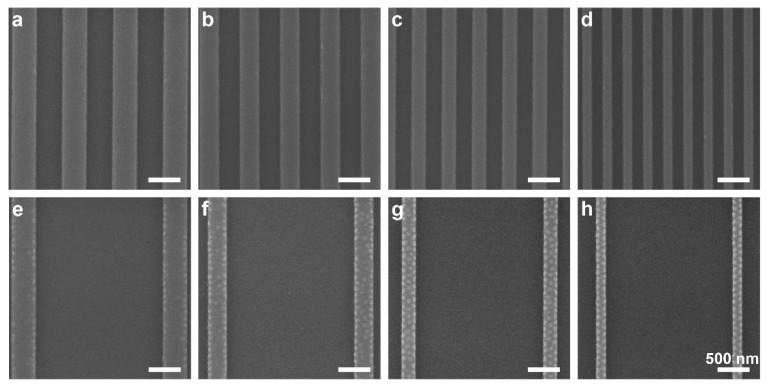
SEM images of films cast on substrates featuring 38 nm-deep trenches and equal plateau and trench widths: (**a**) 400 nm; (**b**) 320 nm; (**c**) 240 nm; (**d**) 160 nm. Nominal film thickness is 23 nm. Bright and dark stripes correspond to the plateaus and trenches, respectively. (**e**–**h**) SEM images of the same trench depth (38 nm) and respective plateau widths, spaced by 2 µm-wide trenches. All scale bars represent 500 nm.

Images of the deepest trench depths studied, 38 nm, show another interesting trend (Figure 5). Whereas symmetric plateau and trench dimensions show a completely patternless morphology over all areas of the features (Figure 5a–d), using wider trenches leads to the formation of a dot pattern on the plateaus (Figure 5e–h). The coverage of the dot pattern increases as the plateau widths decrease. Interestingly, we find that the deposited film is thinner both on the plateau and in the trench when the film is cast on a substrate featuring wide trenches than for similar plateau widths with narrower trenches (see Supplementary Table S1). This indicates that less material remains on the patterned area during spin coating when the plateaus are widely spaced, which is attributed to the influence of feature density, as has been previously reported [41,42].

## 4. Discussion

The results presented above show that the local film thickness alone cannot account for the observed morphologies, and reveal a clear influence of the trench depth and plateau and trench widths on the morphologies obtained on the plateaus and in the trenches, as well as on the formation of dual patterns. Interdependence between the dimensions of the topographic features is also evident, and makes the analysis challenging.

A telltale sign on the reason for the influence of the topographic feature dimensions on the local morphologies comes from a close inspection of the edges of the plateaus in films cast on 31 nm-deep trenches (Figure 4). We observe that the edges are decorated by PMMA dots over most of their length, even in plateaus that feature only partial coverage of dots. Similar behavior is observed in films cast on 38 nm-deep trenches, where the plateaus are separated by wide trenches (Figure 5e–g). The high density of dots observed on narrow plateaus is thus explained as arising from the overlapping influence of both plateau edges, which cover the entire plateau area. Hence, we propose that domain nucleation starts at the plateau edges [6,17]. In the case of the wide plateaus (e.g., Figure 4a; *w*_pl_ = 640 nm ≈ 8*L*_0_), the plateau edges are decorated with PMMA dots on the major part of their length, and these edge domains seem to template the formation of additional dots toward the center of the plateau. In comparison, the narrow plateaus (e.g., Figure 4d; *w*_pl_ = 160 nm ≈ 2*L*_0_) are almost entirely covered with hexagonally packed PMMA dots, because at this width, the entire span of the plateau is under the range of influence of the edges.

The complete absence of PMMA features at the surface of the film cast on 38 nm-deep trenches with similar plateau/trench widths (Figure 5a–d) suggests that the existence of the plateau edges in this system is insufficient to induce the nucleation of PMMA domains. As the local film thicknesses at the center of the plateaus and trenches are rather similar for both 31 and 38 nm-deep trenches (when comparing patterns with identical lateral dimensions), another variable that could explain the difference in morphological behavior is the curvature of the film profile at the plateau edge, which may lead to local frustration of the chains [43]. As we demonstrate hereafter, this variable is directly related to the dimensions of the topographic features.

**Figure 6:**
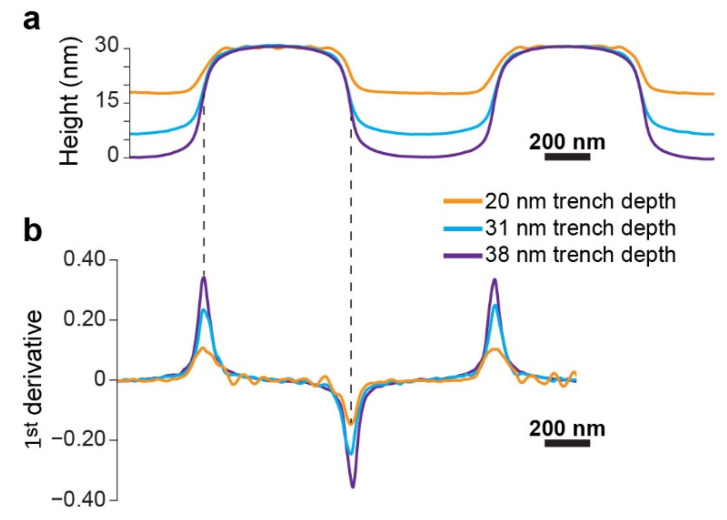
(**a**) Overlay of the average SFM film profile for different trench depths for plateau/trench widths of 640/640 nm. Film profiles were offset to overlap at the top of the plateau film to facilitate visual comparison. (**b**) First derivative curves of the film profiles shown in (**a**).

Figure 6a shows an overlay of the profiles of the film surface for various trench depths measured by scanning force microscopy (SFM), which reveals that the increase in trench depth significantly changes the curvature of the film above the plateau edges. The same trend is general and is also observed with substrates featuring other plateau/trench widths, including asymmetric combinations (Supplementary Figure S2). Figure 6b shows the first derivative curves corresponding to the height profiles shown in Figure 6a. The peaks of the first derivative curves, which represent the steepest slopes (both positive and negative) in the film profile near the plateau edges, increase in magnitude with increasing trench depth. Hence, we set to investigate how the local morphology relates to the film curvature (represented by the steepest slopes in the film profile).

The shape and film thickness of a film spin-coated over a topographic substrate are determined by a number of forces and the interplay between them. Centrifugal force, capillary forces, and gravity cause flow in the spin-cast solution and are counteracted by viscous forces. As the casting solvent evaporates, the film undergoes further shrinkage and vitrifies [44,45,46,47]. The resulting films are characterized by non-planar contours over the topography, where the height contrast between plateau and trench areas is smaller for narrow features than for wider features [41,42,47,48]. In relatively thick films, it has been previously shown that the film profile is insensitive to small changes in trench depth [41]. The works cited herein, however, were all performed on homopolymer films, which lack an internal, microphase-separated structure. A theoretical study on thin BCP films cast on topographic substrates and annealed indicates that different domain orientations may co-exist [49]. To the best of our knowledge, however, the influence of the film surface curvature on the film morphology has not been previously explored.

**Figure 7:**
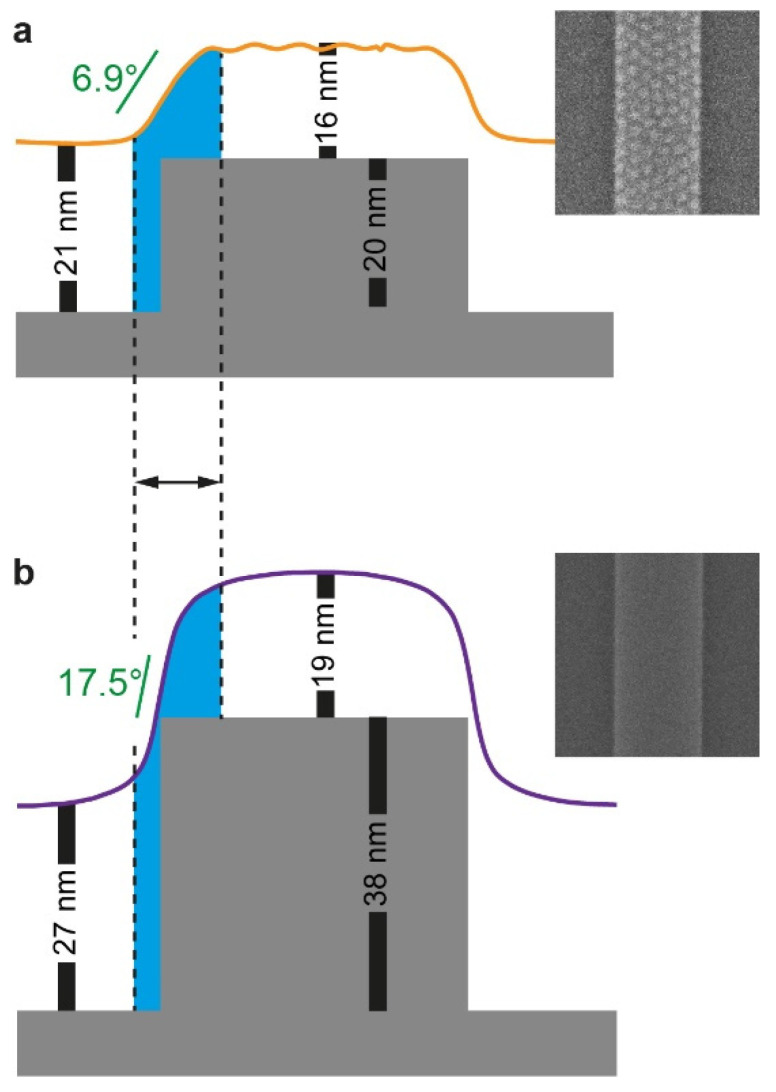
Schematic illustration demonstrating the correlation between the profile slope at the plateau edge and the pattern on the plateau. The blue shading highlights the zone of thickness frustration. The SFM film profiles (from Figure 6a) of 22–23 nm-thick films are overlaid on the schematics of the corresponding substrate featuring 640/640 nm trench/plateau widths with different trench depths: (**a**) 20 nm; (**b**) 38 nm. Height scale is exaggerated; the angular values of the steepest slopes at the plateau edges, calculated from the peak values in the curves in Figure 6b, are provided for reference. Insets show the corresponding patterns (Figure 2e,i).

Although the reason for the correlation between the film curvature and the local morphology is unclear, we speculate that a more gradual change in the film profile across the plateau edge (i.e., a milder slope) may lead to larger areas along the edge of the plateau in which the polymer chains are conformationally frustrated by the local variations in film thickness (Figure 7a). This frustration may be alleviated by orienting the domains normal to the substrate, in analogy to films featuring thicknesses that are slightly incommensurate with the bulk periodicity of the BCP cast on neutral substrates [50,51]. The vertically oriented domains formed at the edge of the plateau further template the formation of additional vertically oriented domains, and this templation further propagates into the center of the plateau as we suggested above. Conversely, when the profile slope at the edge of the plateau is too steep (i.e., in the deep trench case), the zone of frustration across the edge of the plateau may be too narrow to accommodate the width of a fully developed, vertically oriented PMMA domain, especially when the film is nominally thinner than the trench depth (Figure 7b). In this case, the edges cannot function as sites for nucleation of vertically oriented domains. Additionally, the steep profile change, in this case, makes the film on the plateaus thick enough to accommodate a lying lamella, even in the immediate vicinity of the plateau edge. Both effects cause films cast on substrates featuring deep trenches to appear featureless not only in the trenches but also on the plateaus.

**Figure 8:**
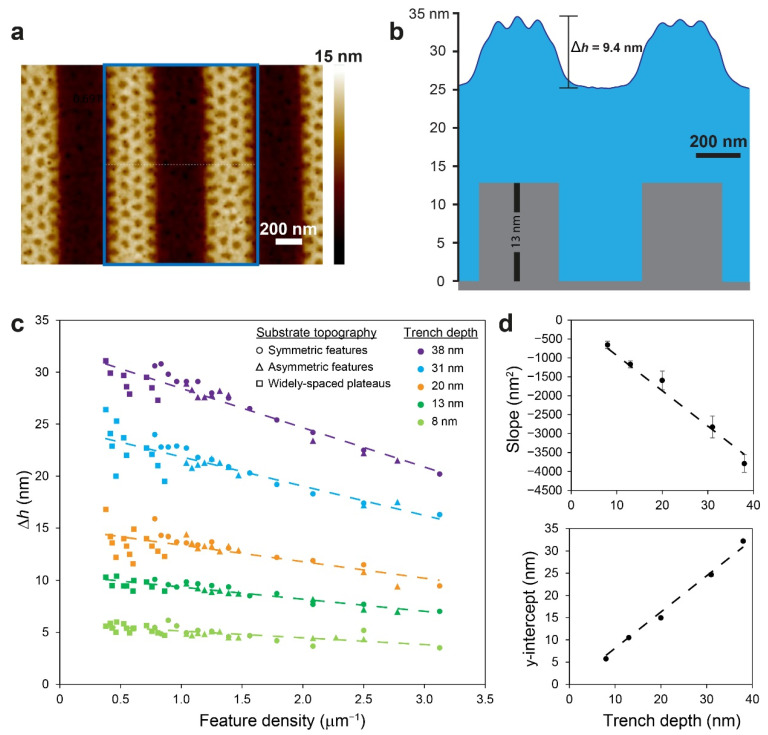
(**a**) A typical SFM scan used for measurement of the height contrast between the film on the plateau and the film in the trench (*d* = 13 nm; *w*_pl_ = *w*_tr_ = 360 nm). (**b**) The corresponding step analysis of the film profile for the box marked in (**a**) overlaid on a cartoon representing the substrate topography. (**c**) Dependence of height contrast on the topographic feature density. Dashed lines indicate the linear regression results. (**d**) Dependence of the slopes and intercepts of the data in (**c**) on trench depth.

It should be noted that the slope of the film at the plateau edge could be approximated by the height contrast of the film between the plateau and trench regions that is directly measured by SFM scanning, ∆*h*. Interestingly, for a given trench depth, *d*, we find linear relationships between the height contrast and the density of topographic features (defined as (*w*_pl_ + *w*_tr_)^−1^) for all combinations of trench and plateau widths, both symmetric and asymmetric, including widely spaced plateaus (Figure 8a–c). This dependence becomes more accentuated for deeper trenches, which means that a deeper topography offers more versatility because their film profile changes over a larger range with the topographic feature density (as was observed for the 31 and 38 nm feature depths). Additionally, Figure 8d reveals a linear dependence of the slopes and intercepts shown in Figure 8c on trench depth. Combining these trends yields the following equation (where all variables are given in nm):

(1) $$\Delta h=\left(0.8140-\frac{93.44}{w_{\mathrm{pl}}+w_{\mathrm{tr}}}\right)d.$$

Decoster et al. found a similar dependence (∆*h* = *pd*, where *p* quantifies the remaining topography after the ‘planarization’ of topographic features by the spin-coated material) [41]. Our analysis shows that with our PS-*b*-PMMA copolymer, the extent of planarization is at least 18.6% (for wide plateaus and/or trenches), and it further increases in a linear fashion with increasing density of topographic features (i.e., decreasing plateau and/or trench widths), where complete planarization is expected at *w*_pl_ + *w*_tr_ ≈ 115 nm (which is outside the feature density range in this study). More importantly, this analysis provides an easy method for estimating the expected height contrast of the PS-*b*-PMMA film used in this study, given the dimensions of the topographically patterned substrate on which it is cast. We believe Equation (1) represents a general relationship that should be applicable to other polymer films as well, where the coefficients depend on the characteristics of the polymer used and the nominal thickness of the film.

**Figure 9:**
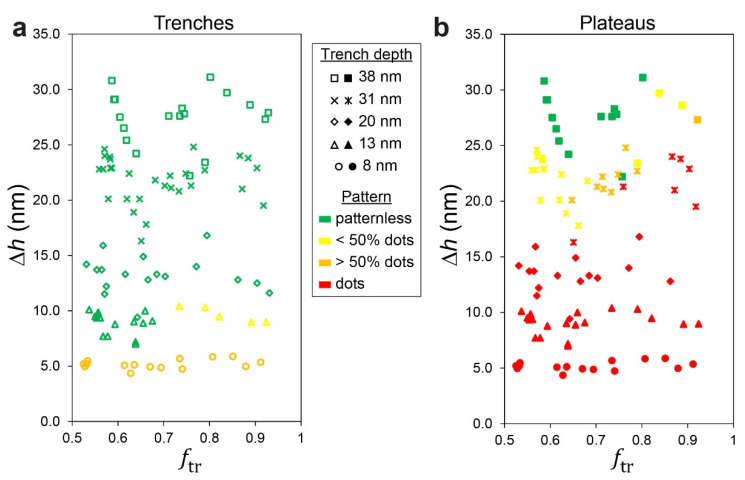
Phase diagrams of the morphologies of lamellar PS-*b*-PMMA cast over topographically patterned substrates for a constant nominal film thickness of 22–23 nm: (**a**) patterns that develop in the trenches; (**b**) patterns that develop on the plateaus.

The relation between the height contrast and the local morphology can be easily observed when separate phase diagrams corresponding to morphologies evolving on the plateaus and trenches are plotted against the volume fraction of the BCP film that is deposited in the trench, *f*_tr_ (Figure 9). The values of *f*_tr_ were approximated from the widths of the plateaus and trenches, *w*_pl_ and *w*_tr_, and the corresponding local film thicknesses, *h*_pl_ and *h*_tr_, determined from SFM scans of scratched samples (see the Experimental section for details):

(2) $$f_{\mathrm{tr}}=\frac{h_{\mathrm{tr}}w_{\mathrm{tr}}}{h_{\mathrm{tr}}w_{\mathrm{tr}}+h_{\mathrm{pl}}w_{\mathrm{pl}}}.$$

The phase diagrams enable us to semi-quantitatively distinguish between the pattern of dots at different levels of coverage (sparse, partial, and complete) and a patternless appearance. A clear dependence on the height contrast is observed in both diagrams, where very high contrasts (top parts of the diagrams; Δ*h* > 25 nm) lead to a patternless appearance of the entire film (as shown in Figure 5a–d); very low contrasts (bottom parts of the diagrams; Δ*h* < 12 nm) lead to dots all over the film (with complete coverage on the plateau and partial coverage in the trenches, as shown in Figure 2a,b); and dual morphology in the central parts of the diagrams (12 nm < Δ*h* < 25 nm), where the slope of the film profile at the plateau edges is neither too steep nor too shallow (as shown, for example, in Figure 3). The difference between the behaviors of the film in the trench from the film on the plateau is also evident. The employment of the volume fraction of the film in the trench as the horizontal ordinate also provides reasonable separation between regions that feature complete or partial coverage of dots on the plateaus as well as patternless vs. sparse dots in the trenches, and their dependence on the trench width (as shown in Figure 4 and Figure 5). Although the employment of the volume fraction of the film in the trench is only empirical, it is reasonable because it takes into account not only the lateral dimensions of the topographic features in the substrate but also the local film heights, which obviously influence the local morphology. Specifically, *f*_tr_ gives a large weight to wider trenches (because the capillary forces acting during spin coating direct more material to deposit into the trench) [41], which is consistent with the observation made above regarding the influence of wide trenches on the pattern that develops on the plateaus (Figure 5e–h). Indeed, using other reduced parameters, such as feature density ((*w*_pl_ + *w*_tr_)^−1^), duty cycle (*w*_pl_/(*w*_pl_ + *w*_tr_)), or effective plateau width (*w*_pl_ *w*_tr_/(*w*_pl_ + *w*_tr_), which emphasizes the influence of isolated plateaus or trenches) [42], yielded poorer separation between different morphological regions in the phase diagrams (not shown) and emphasized the need to account for local film thicknesses in the analysis.

**Figure 10:**
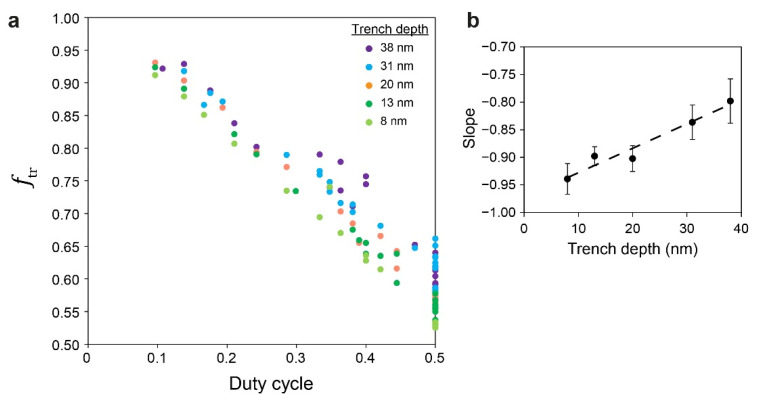
(**a**) Dependence of fraction of BCP in the trench on the duty cycle (see Supplementary Figure S3 for the separate curves). (**b**) Dependence of the slopes of the data in (**a**) on trench depth.

Making the phase diagrams useful as a prediction and planning tool requires an easy way to also estimate the value of *f*_tr_ for a given system from the given dimensions of the topographic features. Interestingly, in analogy to the estimation of ∆*h* from its dependence on topographic feature density, we found linear correlations between *f*_tr_ and the duty cycle (Figure 10a) and linear dependence of their slopes on trench depth (Figure 10b). These relationships enabled us to derive the following equation (where all variables are given in nm):

(3) $$f_{\mathrm{tr}}=1+\left(0.005672d-0.9602\right)\frac{w_{\mathrm{pl}}}{w_{\mathrm{pl}}+w_{\mathrm{tr}}}.$$

## 5. Conclusions

This work highlights the crucial role that the curvature of the film at the plateau edges plays in the development of local morphologies on the plateaus and in the trenches. This insight adds to the known dependence of the local morphology on slight variations in film thickness, which are afforded by the topographic features, and that may lead to the controlled creation of dual patterns [36,37]. We suggest that the gradual variation in film thickness at the plateau edges leads to the formation of perpendicularly oriented domains along the edges, which further template the formation of additional domains that may cover the entire plateau.

An analysis of multiple samples featuring different trench depths and different combinations of plateau and trench widths yielded separate phase diagrams for the behavior of the films confined in the trenches and the films deposited on the plateaus. These phase diagrams display different regions of morphological behavior, where the morphologies are ascribed to two variables: the height contrast between the plateau and trench, which is correlated with the film curvature at the edges of the plateaus and is measured by scanning force microscopy, and the volume fraction of the film that resides in the trenches, which takes into account the local film thicknesses. We further show that the height contrast and the fraction of film in the trench linearly depend on the density of topographic features and the duty cycle, respectively. Further linear dependence of both parameters on the trench depth allowed us to derive two equations that enable us to predict the height contrast and fraction of film in the trench from the dimensions of the topographic features.

We believe that the linear dependencies we discovered are universal and could be generalized to other block copolymer systems and cast thicknesses (with different coefficients that relate to the type of polymer used and the cast thickness). Thus, providing the phase diagrams of the plateaus and trenches for a given block copolymer film in its cast (nominal) thickness allows one to predict the morphology that would be observed in each region of the film for any topographic feature. Alternatively, it allows one to plan the topographic feature dimensions that would lead to the desired morphology. Such an ability has the potential to advance the fabrication of devices that require the combination of different structures on the same chips, such as metasurfaces and photonic integrated circuits.

## Data Availability

Not applicable.

## Supplementary Materials

The following supporting information can be downloaded at: https://www.mdpi.com/article/10.3390/polym14122377/s1, Figure S1. Overlay of SFM scans of a bare substrate (gray line) with 38 nm-deep and 640 nm-wide trenches and 320 nm-wide plateaus, and a nominally 23 nm-thick film (blue curve) cast over the same substrate. The distance between the overlaid curves, 23.5 nm, was determined by imaging the film near a scratch that exposed the substrate and enabled the determination of absolute film thicknesses; Table S1. Selected local film thickness measurements for samples with 38 nm-deep trenches; Figure S2. Overlays of the average film profiles for different trench depths, as measured by scanning force microscopy for plateau/trench widths of: (**a**) 640/320 nm; (**b**) 320/320 nm. Film profiles were offset to overlap at the top of the plateau film to facilitate visual comparison; Figure S3. Dependence of fraction of BCP in the trench on the duty cycle for different trench depths: (**a**) 8 nm; (**b**) 13 nm; (**c**) 20 nm; (**d**) 31 nm; (**e**) 38 nm. Dashed lines indicate the linear regression results.
