# Supplementary material for: Dual Block Copolymer Morphologies in Ultrathin Films on Topographic Substrates: The Effect of Film Curvature
Source: Polymers (Basel). 2022 Jun 12;14(12):2377. doi: 10.3390/polym14122377 (PMC9231016; doi:10.3390/polym14122377)
Supplement: Supplementary file 1 [file polymers-14-02377-s001.zip › polymers-1749813-SM.pdf]

polymers

MDPI

Article

Dual Block Copolymer Morphologies in Ultrathin Films on Topographic Substrates: The Effect of Film Curvature

Elisheva Michman, Meirav Oded and Roy Shenhar *

Institute of Chemistry and the Center for Nanoscience and Nanotechnology, The Hebrew University of Jerusalem, Jerusalem 9190401, Israel; elisheva.michman@mail.huji.ac.il (E.M.); meirav.oded@mail.huji.ac.il (M.O.)
* Correspondence: roys@huji.ac.il

Abstract: The ability to create mixed morphologies using easily controlled parameters is crucial for the integration of block copolymers in advanced technologies. We have previously shown that casting an ultrathin block copolymer film on a topographically patterned substrate results in different deposited thicknesses on the plateaus and in the trenches, which leads to the co-existence of two patterns. In this work, we highlight the dependence of the dual patterns on film profile. We suggest that the steepness of the film profile formed across the plateau edge affects the nucleation of microphase separated domains near the plateau edges, which influences the morphology that develops on the plateau regions. Analysis of the local film thicknesses in multiple samples exhibiting various combinations of plateau and trench widths for different trench depths enabled the construction of phase diagrams that unraveled the intricate dependence of the formed patterns not only on the curvature of the film profile but also on the fraction of the film that resides in the trenches. Our analysis facilitates the prediction of the patterns that would develop in the trenches and on the plateaus for a given block copolymer film of known thickness from the dimensions of the topographic features.

Keywords: Block copolymers; Directed self-assembly; Thin films; Hierarchical structures; Patterning

Citation: Michman, E.; Oded, M.; Shenhar, R. Dual Block Copolymer Morphologies in Ultrathin Films on Topographic Substrates: The Effect of Film Curvature. *Polymers* **2022**, *14*, 2377. https://doi.org/10.3390/polym14122377

Academic Editors: Katharina Brassat and Jörg K. N. Lindner

Received: 15 May 2022
Accepted: 7 June 2022
Published: 12 June 2022

Publisher's Note: MDPI stays neutral with regard to jurisdictional claims in published maps and institutional affiliations.

Copyright: © 2022 by the authors. Submitted for possible open access publication under the terms and conditions of the Creative Commons Attribution (CC BY) license (https://creativecommons.org/licenses/by/4.0/).

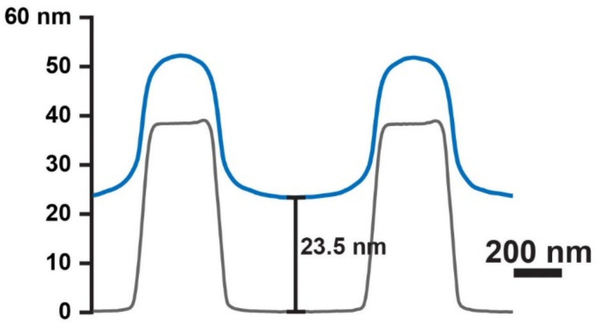

Figure S1. Overlay of SFM scans of bare substrate (gray line) with 38 nm-deep and 640 nm-wide trenches and 320 nm-wide plateaus and a nominally 23 nm-thick film (blue curve) cast over the same substrate. The distance between the overlaid curves, 23.5 nm, was determined by imaging the film near a scratch that exposed the substrate and enabled the determination of absolute film thicknesses.

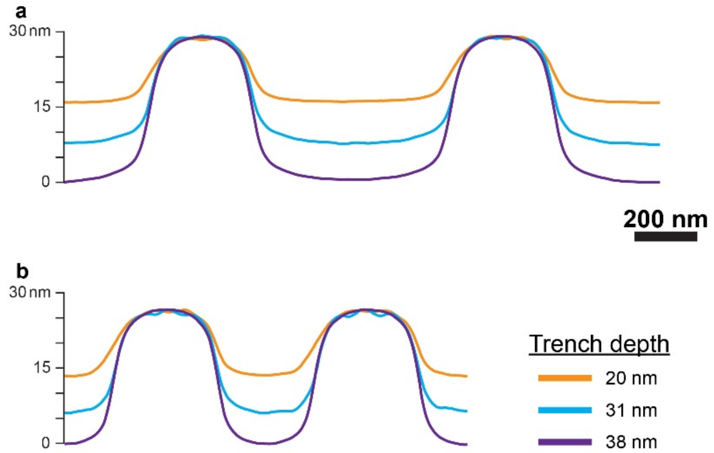

Figure S2. Overlays of the average film profiles for different trench depths, as measured by scanning force microscopy for plateau/trench widths (**a**) 640/320 nm and (**b**) 320/320 nm. Film profiles have been normalized to the top of the plateau film to facilitate visual comparison.

Table S1. Selected local film thickness measurements for samples with 38 nm-deep trenches.

w_{pl} (nm)	h_{tr} (nm)		h_{pl} (nm)	
240	27.5 (w_{tr}=w_{pl})	23.7 (w_{tr}=1.5 µm)	16.2 (w_{tr}=w_{pl})	11.9 (w_{tr}=1.5 µm)
320	27.3 (w_{tr}=w_{pl})	25.7 (w_{tr}=1.5 µm)	17.4 (w_{tr}=w_{pl})	15.5 (w_{tr}=1.5 µm)

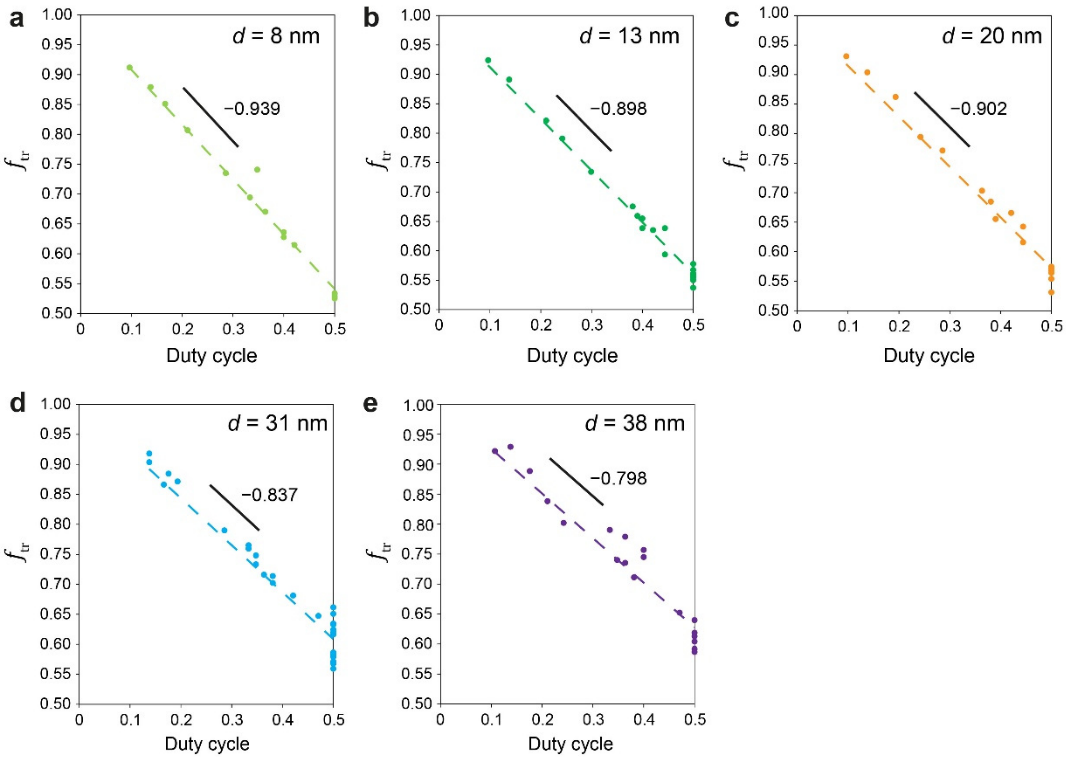

Figure S3. Dependence of fraction of BCP in the trench on the duty cycle for different trench depths: (**a**) 8 nm; (**b**) 13 nm; (**c**) 20 nm; (**d**) 31 nm; (**e**) 38 nm. Dashed lines indicate the linear regression results.
